# “Small Talk” in the Innate Immune System via RNA-Containing Extracellular Vesicles

**DOI:** 10.3389/fimmu.2014.00542

**Published:** 2014-10-29

**Authors:** Susanne G. van der Grein, Esther N. M. Nolte-’t Hoen

**Affiliations:** ^1^Department of Biochemistry and Cell Biology, Faculty of Veterinary Medicine, Utrecht University, Utrecht, Netherlands

**Keywords:** extracellular vesicles, exosomes, microRNA, innate immune system, infection

## Abstract

A newly uncovered means of communication between cells involves intercellular transfer of nano-sized extracellular vesicles (EV), composed of lipids, proteins, and genetic material. EV released by cells of the immune system can play a regulatory role in the induction and suppression of immune responses. These functions may be mediated not only by the bioactive lipids and proteins present in EV but also by EV-associated RNAs. The RNA in EV mainly consists of microRNAs and a large range of other small non-coding RNA species. Since many of these small RNAs have the potential to regulate gene expression, intercellular transfer of these RNAs via EV may cause long-term changes in the function of EV-targeted cells. Several types of innate immune cells release EV that affect innate immune responses and other (patho)physiological processes. Additionally, the innate immune system is influenced by EV released by non-immune cells and EV found in body fluids. In this review, we focus on how EV-associated RNAs contribute to these immune regulatory processes.

## Introduction

The immune system is a complex and highly organized system, consisting of multiple cell types that reside throughout the body. To mount coordinated and effective responses to invading pathogens, it is essential that these different immune cells communicate with each other. Three well-defined mechanisms of intercellular communication are contact-dependent signaling via membrane-bound molecules or gap junctions, paracrine signaling via secreted soluble molecules (such as cytokines and chemokines) that act on nearby cells, and endocrine signaling via hormone secretion into the blood stream to reach distant target cells. Over the last decade, it has become clear that cells also communicate via the release of nano-sized vesicles into the extracellular milieu [reviewed in Ref. (1, 2)]. Consequently, the research interest to unravel the composition and function of such extracellular vesicles (EV) has largely intensified. EV are currently considered to constitute a sophisticated method of information exchange between cells by serving as transport vehicles for a variety of biologically active molecules, including proteins, lipids, and nucleic acids (mainly small RNAs) (2). Intercellular communication via such vesicles can play an important role in regulation of physiological and pathological processes (3). With regard to their role in the immune system, there is steadily accumulating *in vitro* and *in vivo* evidence that EV are functionally transferred between various types of immune cells [reviewed in Ref. (4)]. After introducing general aspects of EV and their supposed role in the immune system, this review will focus on the role of small non-coding RNAs associated to EV in regulation of innate immune responses.

## Extracellular Vesicles for Intercellular Communication

**Figure 1:**
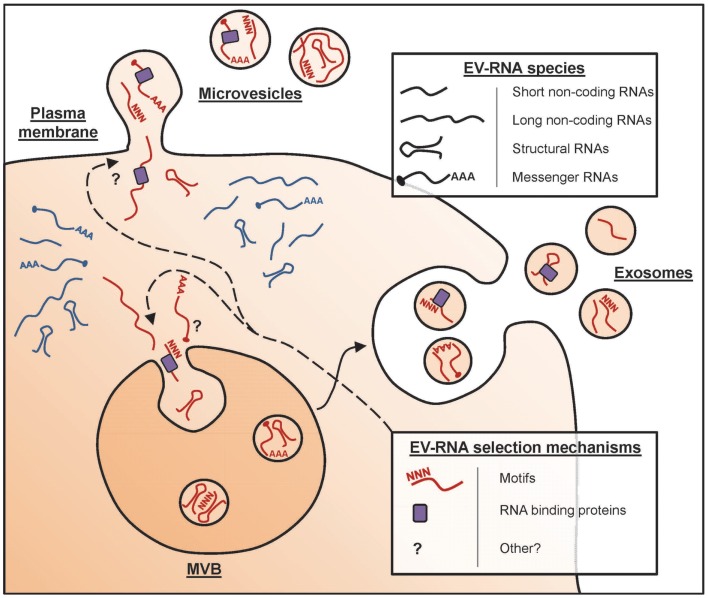
**Formation of RNA-containing EV**. In mammalian cells, subpopulations of EV arise in different subcellular compartments. The different types of EV include exosomes, which are released upon fusion of MVB with the plasma membrane, and microvesicles that pinch off directly from the plasma membrane. Depicted are the RNA species detected in EV, i.e., short-non-coding RNAs (e.g., miRNA and tRNA fragments), long-non-coding RNAs, structural RNAs (e.g., Vault RNA and SRP-RNA), and protein-coding mRNAs. It is currently unknown whether all EV contain RNA and whether differences exist in the RNA content of various EV subpopulations. Various studies indicate that selective RNAs are enriched in EV (red), whereas others are preferentially retained in the cell (blue). This RNA sorting process may depend on specific motifs in the RNA sequence and/or may involve the action of RNA-binding proteins (e.g., the ribonucleoprotein A2B1) in addition to yet unidentified mechanisms.

The collective term “EV” is used for any type of cell-derived vesicle in the extracellular space. A vast array of cell types has been shown to release EV, which can be detected in culture supernatants of *in vitro* cultured cells and in several body fluids, including blood, urine, and milk (5–7). Such EV can have different subcellular origins [Figure 1, and reviewed in Ref. (2)]. EV can be formed as intraluminal vesicles via inward budding from the limiting membrane of late endosomal compartments [multivesicular bodies (MVBs)], which are released into the extracellular space upon fusion of MVBs with the plasma membrane. These EV are commonly referred to as exosomes, and have a diameter of 50–100 nm. Alternatively, EV can be formed by outward budding from the plasma membrane. These vesicles have a wider range of sizes up to 1 μm in diameter and are often referred to as microvesicles. With the currently available technologies, it is not possible to discriminate exosomes from microvesicles. Moreover, EV populations found in cell culture supernatant and body fluids are often heterogeneous in size, morphology, and molecular contents. The overall molecular composition of EV depends on the type, differentiation and activation status of the donor cells, as well as on the conditions under which the vesicles are produced (8). The lipid bilayer that encloses EV protects the luminal message molecules from being degraded in the extracellular environment. However, bioactive lipids and proteins in the EV membrane can also play a role in the targeting of these vesicles to their destination and in signaling to EV-targeted cells. The protein content of EV is characterized by the presence of common EV-associated proteins that are shared among vesicles derived from various different producer cell types. This category of EV proteins includes scaffolding proteins of the tetraspanin family, membrane transport proteins, and cytoskeletal elements [reviewed in Ref. (9)]. In addition, EV also contain producer cell type-specific proteins, such as the major histocompatibility complex class II (MHC II) molecules that are present in EV secreted by antigen presenting cells (APCs) (10). EV-associated nucleic acids mainly consist of small RNAs (see below), which can mediate regulation of gene expression in target cells. Thus, EV can be viewed as multicomponent signaling devices that can modulate the function of a recipient cell upon delivery. Fundamental research in the EV field focuses on unraveling the mechanisms that control their composition and release and on understanding their biological activity. In clinical settings, EV in body fluids are explored as candidate biomarkers for detection and monitoring of diseases. This is based on the idea that the presence and abundance of specific types of EV in body fluids reflect the health or disease status of the tissues they originate from. Furthermore, vesicles derived from dendritic cells (DCs) have been therapeutically applied in clinical trials for tumor vaccination (11).

## EV-Mediated Communication in the Immune System

Immune cell-derived EV have been extensively studied and these EV were shown to play a regulatory role in both innate and adaptive immune responses [reviewed in Ref. (1, 4, 8)]. Generally stated, the functions of EV in the immune system are heterogeneous and correlate with the biological activity of their associated molecules. Immune cell-derived EV can have either immune stimulatory or immune down-regulatory effects (4, 8). Immune activating functions of EV include antigen presentation (by APC-derived EV) and the induction of pro-inflammatory cytokine release (by various immune cell-derived EV). Immune suppression, on the other hand, can be induced by EV from tolerogenic DCs and by apoptosis-inducing T cell EV. EV-mediated functions in the immune system have mostly been demonstrated *in vitro*, whereas the *in vivo* function of EV in various physiological and pathological settings remains ill-understood. However, changes in the blood EV profile observed in systemic inflammatory disorders may indicate a role for EV in these pathologies (12, 13).

Intercellular communication via EV relies on their uptake by recipient cells. Details on the efficiency and specificity of this process are largely unknown. Once released, EV can be delivered to neighboring cells in a paracrine fashion or to interacting cells at specialized cell–cell junctions, such as the immune synapse. Immune synapses are highly organized supramolecular structures formed at the contact area between interacting cells, such as T cells interacting with APCs and NK cells interacting with target cells (14). An immune synapse allows for efficient exchange of signaling or effector molecules, like cytokines and cytotoxic agents. There is evidence that T cell-derived vesicles can be transferred to an interacting APC via immune synapses, indicating that these synapses are also involved in the intercellular transfer of EV (15, 16). Although these studies claim that EV transfer only occurs from T cells to APC, we and others previously demonstrated that transfer of EV between these cells likely occurs in a bidirectional fashion, since EV released from DC could be detected on the surface of T cells (17, 18). Despite the observed transfer of EV via synapses, the abundant presence of EV in culture supernatants indicates that only part of the produced EV are released within the contact area formed between interacting cells.

Besides short-range transfer, EV can also exert long-range effects by gaining access to body fluids. Via these fluids, EV may reach peripheral tissues, allowing the transfer of messages between cells at a distance. Whereas for locally delivered EV, the targeting specificity may be controlled by directional release of vesicles into a confined space, it is difficult to envision directed targeting of EV circulating throughout the body. For systemically disseminated vesicles, targeting specificity may rely more on selective responsiveness of the recipient cells, meaning that only a restricted subset of cell or tissue types may be able to acquire and respond to the vesicle-enclosed messages. Target cell binding has been suggested to depend on tetraspanins and integrins on the surface of EV (19–21). For EV targeting to lymphoid cells and tissues, several interaction molecules were proposed to be involved. LFA-1 binding to ICAM-1 is important for binding of DC-derived EV to both activated T cells (20) and to CD8^+^ DCs in lymph nodes (19). The sugar-binding protein sialoadhesin (CD169) on macrophages in the subcapsular sinus and medulla of lymph nodes and the splenic marginal zone can bind to α2,3-linked sialic acids on the surface of B cell-derived EV, suggesting that these cells are involved in controlling lymphoid organ access of these EV (22). In addition, opsonization of DC-derived EV by complement C3 fragments or lactadherin (MFG-E8) was proposed to promote vesicle capture by DCs (23, 24). The complement receptor CD21 present on marginal zone B cells (MZBs) and follicular dendritic cells (FDCs) in lymphoid organs may be involved in efficient capture of these opsonized EV within these organs (24, 25). Taken together, it is likely that selective target cell binding of EV is dependent on specific combinations of interaction molecules on both EV and target cells.

## EV are Enriched in Non-Coding RNAs

In recent years, many reports have emerged on the presence of RNA in EV released by several different cell types. The majority of EV-associated RNA consists of small RNA species (<200 nt), but selected mRNAs and long-non-coding RNAs (lncRNAs) have also been detected [Figure 1, Ref. (26)]. The first data on functional transfer of EV-associated RNA to target cells were reported in 2007 by Valadi et al., who showed that murine mRNA transferred via EV could be translated in recipient cells of human origin (27). The horizontal transfer of genetically encoded messages via EV is now recognized as a newly identified means of intercellular communication.

Based on the observed abundance of small RNAs in EV, many studies have focused on characterizing the microRNA (miRNA) content of EV. Comparison of the miRNA profiles of EV and their donor cells have indicated that some miRNAs are preferentially released via EV (such as miR-451 and miR-150), whereas others (such as miR-218) are retained in the cell (28–30). Moreover, populations of EV-associated miRNAs often contain both common and donor cell type-specific miRNAs. For example, miR-451 and miR-150 are preferentially exported in EV from different cell types, including endothelial cells, tumor cells, and mesenchymal stem cells, whereas miR-142-3p is preferentially exported by epithelial cells, but not by immune cells such as DC and T and B lymphocytes (29). These distinct vesicle and cellular miRNA profiles indicate that the nucleic acid composition of EV does not merely reflect the cellular pool of nucleic acids. Instead, RNA molecules appear to be selectively incorporated into EV. Several studies addressed which mechanisms control selective packaging of RNA into EV. Selectivity in miRNA incorporation is not the result of co-segregation with complementary mRNA sequences, as illustrated by the underrepresentation of miRNA-repressible mRNA transcripts in vesicles (31, 32). It has been suggested that the miRNA processing machinery could aid in the loading of miRNA molecules into exosomes. The RNA-induced silencing complex (RISC) is involved in miRNA processing from precursor forms and facilitates miRNA-mediated degradation of target mRNAs. This multiprotein complex was observed to physically colocalize with MVBs from which exosomes arise (31, 33), suggesting the possible involvement of RISC in miRNA incorporation into EV. More direct evidence was provided for the involvement of RNA motifs and RNA-binding proteins in packaging of selective RNAs into EV (Figure 1). Studies on glioblastoma cell-derived EV revealed that not a single motif but combinations of multiple motifs were specifically present in EV-associated RNAs (26). In EV released by T cells, short consensus sequence motifs were found in miRNAs that could control their sorting into EV (34). These motifs bound to the heterogeneous nuclear ribonucleoprotein A2B1 (hnRNPA2B1), and this protein was shown to drive loading of the specific miRNAs into EV (34). Whether these findings extend to EV from other cell types and whether there are more motifs that target miRNAs for inclusion in EV remains to be determined.

Besides mRNA and miRNA, EV, also contain other RNA species. We recently characterized the small RNA (<70 nt) contents of immune cell-derived EV by deep sequencing. These vesicles appeared to contain a large variety of small non-coding RNA species. Besides relatively small numbers of miRNAs, we detected pervasive transcripts or cleavage products that annotated to protein-coding regions, repeat sequences, or structural RNAs [RNAs in nucleoprotein complexes performing basic cellular functions, such as signal recognition particle (SRP)-RNA and Vault RNA] (35). Research on small non-coding RNAs is a quickly emerging field and large numbers of non-coding RNA biotypes have recently been discovered in cells (36). Many of these small RNAs represent degradation-like fragments of more abundant RNA species, such as transfer RNAs (tRNA) and mRNAs (36), and are thought to exert gene regulatory functions (37, 38). Several of the small non-coding RNA species found in our immune cell-derived EV, among others the small tRNAs and small Vault RNAs, have also been detected in EV released from neuronal cells and in EV found in urine and blood plasma (39, 40). Although inclusion of these small non-coding RNAs seems common in a variety of EV types, it is currently unknown whether they are functionally important in EV-targeted cells.

In addition to small RNA species, also lncRNAs have been identified in EV. Although the functions of most lncRNAs are as yet unclear, some lncRNAs have been identified as strong regulators of transcription, translation, and epigenetic modifications (41). Recent data indicate that specific lncRNAs with low expression levels in cells (e.g., HOTAIR) were highly enriched in EV (42). Evidence for the functional relevance of lncRNAs in EV came from studies on hepatocellular cancer (HCC) cells (43, 44). A specific lncRNA highly enriched in HCC-derived EV (linc-ROR) was shown to be involved in TGFβ-mediated resistance to chemotherapy (44), indicating a role for EV-associated lncRNA in promoting tumor growth. Taken together, the small and lncRNAs represent a group of regulatory RNAs that can contribute to modulatory effects of EV beyond the activity of miRNAs.

Besides thorough characterization of the RNA content of EV, several studies addressed whether EV-associated RNAs are functionally active when transferred to target cells. EV-mediated delivery of miRNAs by immune cells was shown to downregulate target gene expression in recipient cells (15, 45, 46). In these studies, EV derived from cells overexpressing a miRNA were delivered to recipient cells transfected with a luciferase reporter placed after the 3′UTR of the miRNA-repressible mRNA target. In a small number of recent studies, functional relevance of EV-mediated transfer of *endogenously* expressed miRNAs to target cells was reported (see below). However, no research strategies are available yet to indisputably demonstrate functional EV-mediated transfer of RNAs *in vivo*. Further development of experimental tools is needed that make it possible to specifically interfere with EV release without affecting other vesicle-mediated processes inside the cell. This requires more knowledge on mechanisms behind vesicle secretion, cargo loading, and delivery of enclosed messenger molecules to target cells.

## Role of RNA-Containing EV in the Innate Immune System

### EV in the innate immune system

With regard to the role of EV in the immune system, a large number of studies focused on EV released by cells of the adaptive immune system and this work has been extensively reviewed (4, 8). The involvement of EV in regulation of innate immunity is now increasingly being studied [reviewed in Ref. (8, 47)]. Several types of innate immune cells release EV that affect innate immune responses and other (patho)physiological processes. Additionally, the innate immune system is influenced by EV released by non-immune cells and EV of unknown origin found in body fluids. Macrophages, mast cells, granulocytes, and NK cells are innate immune cells known to release EV that contribute to regulation of immune responses. Mast cells release EV in addition to the release of inflammatory mediators and proteases present in secretory granules (48). These EV contain MHC class II and specific types of heat shock proteins, and were shown to play a role in antigen presentation, maturation of DC, and induction of Th1 responses. Immune activating effects were also observed for EV from bacterially infected macrophages (49), which can activate neighboring macrophages and induce DC maturation. Pro-inflammatory granulocytes, on the contrary, release EV that can downregulate the inflammatory response, e.g., via suppressing the production of pro-inflammatory cytokines by macrophages and immature DC (50).

Insight into the mechanisms behind modulation of innate immunity by EV is limited. Nevertheless, a few studies have reported on a role for EV-associated RNAs in this process. Here, we present an overview of studies that provide proof for functional transfer of EV-associated RNAs in the context of innate immune responses (Table 1). Included are reports on (1) RNA-mediated effects of innate immune cell-derived EV on various (patho)physiological processes and (2) RNA-mediated effects of non-immune cell-derived EV on the innate response. Studies in which EV producer cells were transfected with exogenous miRNA and recipient cells with the corresponding miRNA-reporter construct were not included in Table 1, because of the limited physiological relevance of such a set-up.

**Table 1:** **EV-RNA-mediated functions in the innate immune system**.

Source of EV	EV-RNA type	RNA-mediated effect	Reference
**INNATE IMMUNE CELLS**			
Mast cells	Total RNA	Protection of recipient cells from oxidative stress (indirect proof)	(51)
Monocytes (THP-1)	miRNA-150	Transfer to endothelial cells induced angiogenesis	(52, 53)
Macrophages	miRNA-223	Macrophage differentiation	(46)
	miR-142 and miR-223	Inhibition hepatocarcinoma cell proliferation	(57)
**NON-IMMUNE CELLS**			
Hepatocarcinoma cells	let-7b	Attenuation of inflammatory response in macrophages by targeting IL-6	(58)
Lewis lung carcinoma cells	miR-21 and miR-29a	Triggering of TLR7 (murine) and TLR8 (human), inducing pro-metastatic inflammatory responses	(59)
Various cancer cell lines	EV-associated miRNAs	Triggering of TLR only by tumor-derived (and not by monocyte-derived) EV-RNA	(60)
**VIRUS-INFECTED CELLS**			
EBV-infected tumor B cells	EBV-encoded miRNA miR-BART15	Inhibition of the NLRP3 inflammasome in non-infected monocytic cells	(69)
HCV-infected hepatocytes	Full length HCV RNA	Triggering of pDC IFNα production	(70)
LCMV-infected cells	LCMV RNA	Triggering of pDC IFNα production	(71)
**PARASITES**			
*Trypanosoma cruzi*	tRNA halves (tsRNA^Thr^)	Regulation of genes involved in cell defense and immune responses against pathogens	(78)

### Genetic regulation of innate immune responses by EV-associated miRNAs

As mentioned previously, the earliest studies on EV-RNA-mediated effects concerned mast cells that transferred EV containing miRNAs and mRNAs to target cells (27). The physiological function of EV-associated RNA released by mast cells was addressed a few years later, by investigating the role of EV produced by mast cells exposed to oxidative stress (51). These EV were shown to transfer tolerance to oxidative stress to recipient cells and this function could be inhibited by RNA-degrading UV-light. This suggests that the observed protective effect could be mediated by EV-associated RNA.

Several lines of evidence indicate that EV released by monocytes/macrophages mediate functional transfer of miRNAs to various types of target cells. The function of these transferred miRNAs depends on the activation status of the EV producing cell and the type of recipient cell. EV from the monocytic cell line THP-1 were shown to functionally transfer the angiogenesis regulating miR-150 to the human microvascular endothelial cell line HMEC-1 (52, 53). By using miRNA interference strategies in both the donor and the recipient cell, it was shown that EV-mediated transfer of miR-150 enhanced tube formation of recipient endothelial cells, suggesting that this EV-associated miRNA acts in a pro-angiogenic fashion. Two studies report that EV released by macrophages mediate functional transfer of miR-223, which regulates myeloid cell proliferation/differentiation and tumor development (54–56). Ismail et al. showed that EV release was increased upon differentiation of monocytes into macrophages and that the macrophage-derived EV induced cellular differentiation in targeted monocytes (46). The authors demonstrated that endogenous miR-223 released via EV by macrophages was functional in miR-223-negative target cells transfected with a miRNA-reporter vector. In addition, treatment of monocytes with miR-223 antagomiR was shown to reduce differentiation of these cells. Although evidence for causation was not provided, these data give a strong indication that the EV-induced effects on monocyte differentiation were caused by EV-mediated transfer of miR-223. The enrichment of miR-223 in macrophage-derived EV was confirmed by Aucher et al. (57). Via these EV, miR-223 could be efficiently transferred to hepatocarcinoma cells. Using antagomiRs and miRNA-sponges, strong evidence was provided that the antiproliferative effect of these EV on hepatocarcinoma cells was caused by the EV-associated miR-223. Conversely, EV released by hepatocarcinoma cells were also shown to affect innate immune responses. EV released by these cells contained miRNA let-7b, and transfer of this miRNA to macrophages led to a decrease in the release of the pro-inflammatory cytokine IL-6 (58).

### Toll-like receptor activation by EV-associated miRNAs

Recent data indicate that, besides gene regulatory effects of miRNAs enclosed in EV, specific miRNAs released by tumor cells can act as ligands for toll-like receptors (TLR) (59). Tumor-derived miR-21 and miR-29a were shown to bind to TLR8 (in human beings) and TLR7 (in mice) and activate these receptors in immune cells, leading to secretion of pro-metastatic inflammatory cytokines. Interesting in this respect is the recently published finding that specific EV-associated miRNAs from tumor cells, but not those from non-cancerous leukocytes, can activate TLRs (60). A potential explanation for these data is that endogenous miRNAs from cancer cells, in contrast to miRNAs from healthy cells, are not modified in such a way that TLR activation is avoided. These modifications could for example include 2′-*O*-methyl modification, which is known to act as an antagonist of TLR7/8 (61). This adds another layer of complexity to the functional consequences of intercellular transfer of EV-associated RNA.

### Regulation of innate immune responses by viral RNA-containing EV

Microbial infections can have dramatic effects on the number and function of EV released by cells. EV derived from pathogen-infected cells often contain microbial products that may be recognized by immune cells. Bacterially infected macrophages, for example, show increased secretion of EV that induce DC maturation and activation of neighboring macrophages (62). Several types of viruses also interfere with the release and composition of EV from infected cells [reviewed in Ref. (63, 64)]. For viruses, the interference with EV composition and release can lead to enhanced or suppressed propagation of the virus and/or to modification of the host’s antiviral immune response.

Several studies demonstrated the presence of viral RNA in EV from virus-infected cells and a role of this RNA in regulating the innate immune response (Table 1). Epstein–Barr virus (EBV) is one of the viruses known to interfere with the release of EV. This human herpes virus establishes a latent infection in B lymphocytes and epithelial cells, and EV released by the infected cells contains the EBV latent membrane protein 1 (LMP1) protein. This protein is delivered to surrounding uninfected cells (65), and plays a role in immune evasion. Additionally, EV derived from EBV-infected cells contain EBV-encoded mature miRNAs from BHRF1 and BART, which are two gene clusters of the viral genome (66, 67). The expression patterns of these viral miRNAs are linked to the viral latency stage. Using recipient cells transfected with a miRNA-reporter construct, it was shown that the EV-associated EBV–miRNAs BHRF1-3 and miR-BART15 can be functionally transferred and mediate translational repression of target genes in recipient cells (66, 68, 69). Interestingly, the EBV-encoded miRNA miR-BART15 specifically targets NLRP3, which is a component of the inflammasome, a multiprotein complex that initiates inflammatory processes including production of pro-inflammatory cytokines IL-1β and IL-18 (69). EV-associated miR-BART15 was found to be taken up by monocytic THP-1 cells and the NLRP3 and IL-1β protein expression was decreased in EV-targeted cells (69). These findings, combined with data that transfection of EBV miR-BART15 into THP-1 cells reduced endogenous NLRP3 protein levels, point to a role for EV-associated miR-BART15 in regulating inflammatory responses. In conclusion, the combined transfer of LMP1 and viral miRNA via EV to nearby immune cells likely plays an important role in suppressing immune activation in latent stages of EBV infection.

Besides EBV-infected cells, also hepatitis C virus (HCV) infected cells release EV containing viral RNA. Hepatocytes infected with HCV incorporate full length viral genomic and subgenomic RNA sequences in EV (70). Upon delivery of these EV to non-permissive plasmacytoid DC (pDC), a TLR-7-dependent interferon response was elicited. Similarly, LCMV-infected cells were shown to release LCMV RNA-containing EV that could mediate IFNα production in pDC (71). This EV-mediated mechanism could either serve as a viral strategy to prevent immune recognition or as a host strategy to induce an unopposed antiviral response in neighboring cells in which the virus is unable to replicate.

### RNA in microbe-derived EV

Recent evidence indicates that bacteria and parasites also release EV. These vesicles are not only essential for microbial physiology but can also impact host–microbe interactions and exert immune-modulatory effects [reviewed in Ref. (72, 73)]. Both Gram-positive and -negative bacteria species have been shown to release EV. In Gram-negative bacteria, EV that pinch off from the outer membrane are often called outer membrane vesicles [OMVs; reviewed in Ref. (74)]. Several helminth species have also been shown to release EV (73), but mechanisms behind the formation and release of these EV are unknown. Microbial EV often contain antigens and virulence factors, including ligands for TLRs and NOD like receptors (NLR), which can influence the host’s innate immune response to infection.

Which RNA species are present in microbial EV and how the RNA contents vary between EV of different microbes is largely unknown. However, a recent study by Bayor-Santos et al. demonstrates that EV derived from *Trypanosoma cruzi* contain a variety of small non-coding RNAs (75). Although many parasites lack the canonical regulatory small RNA biogenesis pathways, it is thought that parasitic homologs of regulatory small RNAs exist. Interestingly, specific tRNA-derived small RNAs (tsRNA^Thr^) were abundantly present in these EV (75, 76). Recent findings indicate that production of such tRNA fragments (tRFs) is highly regulated and that these fragments can play a role in several biological processes [reviewed in Ref. (77)]. This is exemplified by the role of tRNA halves in inhibition of protein synthesis, gene silencing via association with different Argonaute proteins, and in viral infectivity. Studies on the function of tsRNA^Thr^ in parasite-derived EV showed that target cell incubation with these EV and direct transfection with tsRNA^Thr^ induced regulation of genes involved in host–parasite biology, such as the gene coding for the key inflammatory cytokine CXCL2 (78). The remarkable enrichment of tRFs in both parasite-derived EV and our previously described immune cell EV (35) may point to an EV characteristic that is conserved across various kingdoms of life. Indeed, an inventory of published EV-RNA deep sequences studies indicates that tRF-enriched EV are released from *Trypanosoma cruzi*, immune cells (35), bacteria (personal communication – Dr. P. Wilmes, Luxembourg), and in seminal fluid (79). Additionally, deep sequencing analysis of EV-associated RNA in urine (39), blood plasma (40), and in EV released by neuronal cells (32) and tumorigenic glioma cells (80) were shown to contain sequences mapping to tRNAs. Whether these sequences represent tRFs or full length tRNAs remains to be investigated.

The above mentioned discoveries indicate that, besides miRNAs, other types of small non-coding RNAs can act as ectopic regulators of translation and transcription after release in EV. Future studies are required to assess the molecular mechanisms behind the gene regulatory function of these RNAs. Furthermore, improvement in technologies to purify and characterize EV is required to more reliably discriminate between extracellular RNA enclosed in nucleoprotein complexes versus EV (81), and to compare the RNA content of different EV subpopulations.

## Conclusion

Extracellular vesicles comprise a highly refined system of intercellular communication, which is widely employed by immune cells. EV are unique carriers of information, since signaling molecules and molecules that mediate selective targeting of the transferred messages are combined within a single particle. This allows for tailor-made delivery of molecular messages to designated recipient cells. Different types of short and long non-coding RNA sequences can be present in EV and, at least *in vitro*, can be functionally transferred to recipient cells. EV-mediated RNA transfer by either innate immune cells or non-immune cells can influence innate (antimicrobial) immune responses. The EV-RNA-mediated effects include regulation of pro- or anti-inflammatory cytokine production and cellular differentiation, and enhancement of antiviral interferon responses. The types of EV-associated RNAs involved in these functions are miRNAs, tRFs, and viral RNA. The EV-mediated intercellular transfer of messages encrypted in several types of non-coding RNAs adds an extra level of regulation for inflammatory responses and host–pathogen interactions.

## Conflict of Interest Statement

The authors declare that the research was conducted in the absence of any commercial or financial relationships that could be construed as a potential conflict of interest.
